# An environmental justice perspective on ecosystem services

**DOI:** 10.1007/s13280-022-01812-1

**Published:** 2022-12-15

**Authors:** Jacqueline Loos, Felipe Benra, Marta Berbés-Blázquez, Leah L. Bremer, Kai M. A. Chan, Benis Egoh, Maria Felipe-Lucia, Davide Geneletti, Bonnie Keeler, Bruno Locatelli, Lasse Loft, Barbara Schröter, Matthias Schröter, Klara J. Winkler

**Affiliations:** 1 Faculty of Sustainability, Institute of Ecology, Leuphana University, Universitätsallee 1, 21335 Lüneburg, Germany; 2 Faculty of Sustainability, Social-Ecological Systems Institute, Leuphana University, Universitätsallee 1, 21335 Lüneburg, Germany; 3 Department of Ecosystem Services, Helmholtz Centre for Environmental Research - UFZ, Permoserstraße 15, 04318 Leipzig, Germany; 4 Institute of Biodiversity, Friedrich Schiller University Jena, Dornburger Straße 159, 07743 Jena, Germany; 5 German Centre for Integrative Biodiversity Research (iDiv) Halle-Jena-Leipzig, Puschstraße 4, 04103 Leipzig, Germany; 6 School of Planning, University of Waterloo, 200 University Ave., Waterloo, ON N2L 3G1 Canada; 7 University of Hawai‘i Economic Research Organization, University of Hawai‘i at Mānoa, 2424 Maile Way, Honolulu, HI 96822 USA; 8 Water Resources Research Center, University of Hawai‘i at Mānoa, Honolulu, HI 96822 USA; 9 Institute for Resources, Environment and Sustainability, University of British Columbia, 2202 Main Mall, Vancouver, BC V6T 1Z4 Canada; 10 Department of Earth System Science, University of California, Irvine, 3219 Croul Hall, Irvine, CA 92697 USA; 11 Department of Civil, Environmental and Mechanical Engineering, University of Trento, Via Mesiano 77, 38123 Trento, Italy; 12 Humphrey School of Public Affairs, Twin Cities, Institute On the Environment, University of Minnesota, 301 19th Ave S, Minneapolis, MN 55455 USA; 13 Forests and Societies, Cirad, University of Montpellier, TA C-105 / D, 34398 Montpellier Cedex 5, France; 14 Working Group Governance of Ecosystem Services, Leibniz Centre for Agricultural Landscape Research, Eberswalder Str. 84, 15374 Müncheberg, Germany; 15 Centre for Sustainability Studies (LUCSUS), Lund University, Box 170, 22100 Lund, Sweden; 16 Department for Natural Resource Sciences, McGill University, Macdonald Campus, 21,111 Lakeshore Road, Ste-Anne-de-Bellevue, QC H9X3V9 Canada

**Keywords:** Environmental management, Equity, Pluralism, Recognition, Relational values

## Abstract

Mainstreaming of ecosystem service approaches has been proposed as one path toward sustainable development. Meanwhile, critics of ecosystem services question if the approach can account for the multiple values of ecosystems to diverse groups of people, or for aspects of inter- and intra-generational justice. In particular, an ecosystem service approach often overlooks power dimensions and capabilities that are core to environmental justice. This article addresses the need for greater guidance on incorporating justice into ecosystem services research and practice. We point to the importance of deep engagement with stakeholders and rights holders to disentangle contextual factors that moderate justice outcomes on ecosystem service attribution and appropriation in socio-political interventions. Such a holistic perspective enables the integration of values and knowledge plurality for enhancing justice in ecosystem services research. This broadened perspective paves a way for transformative ecosystem service assessments, management, and research, which can help inform and design governance structures that nourish human agency to sustainably identify, manage, and enjoy ecosystem services for human wellbeing.

## Introduction

The concept of ecosystem services (ES), the benefits humans derive from nature, has evolved over the years including into the more recent concept of nature’s contributions to people (NCP), which comprises the multiple links between the environment and society that underpin human well-being (Díaz et al. 2018). Through a series of large international assessments, such as the Millennium Ecosystem Assessment (MA 2005), The Economics of Ecosystems and Biodiversity Initiative (TEEB), and the Intergovernmental Science-Policy Platform on Biodiversity and Ecosystem Services (IPBES 2019a), the ES concept is now established in environmental and sustainability research as well as in environmental management and governance practice (Daily and Ruckelshaus 2022). ES research has predominantly focused on developing, conducting, and refining tools for identifying and quantifying socio-economic benefits derived from the biophysical environment (Guerry et al. 2015). Many ES assessment efforts have a biophysical or ecological focus (e.g., quantifying tons of carbon sequestered, tons of soil losses avoided, or the effect of vegetation on local temperatures), with fewer assessments including social or economic valuation (e.g., the reduced vulnerability of coastal communities thanks to climate change mitigation, improved agricultural or hydroelectricity production due to reduced soil erosion, or reduced mortality during heatwaves, Chan and Satterfield 2020; Mandle et al. 2020). While there is increasing attention paid to the distributive and procedural equity dimensions of ES (Mandle et al. 2016), many have argued that much of ES research obscures the justice dimensions associated with values and processes of political decision-making that shape ES management and related policies (Jax et al. 2013; Kolinjivadi et al. 2015; IPBES 2022). Thereby, environmental justice research related to ES assessments majorly focuses on instrumental values corresponding to the distributional equity dimension. By extending beyond instrumental values to also include relational and intrinsic values (Díaz et al. 2018, Pascual et al. 2017a, b), however, the concept of NCP better attends to the recognitional justice dimension.

Although not explicitly articulated, ES thinking aligns with the innate goals of sustainable development (Lele et al. 2013), which embodies central notions of inter- and intragenerational justice within the planet’s biophysical limitations over space and time (Schröter et al. 2017; Bennett et al. 2019). This perspective is in line with international goals such as the Sustainable Development Goal 10 "reduced inequalities". Yet, with justice being a normative concept of what is considered to be morally right (Rawls 1971), its perception (Sen 2009), as well as its configuration and implementation, varies according to historical, social, and legislative contexts (He and Sikor 2015). Capturing this plurality of understandings of justice through place-based approaches for inclusive decision-making is a prerequisite not only for ES framings (Pascual and Howe 2018) and management but also for governance structures and processes directly dealing with natural resource management (Nahuelhual et al. 2018).

To this end, more guidance is needed for researchers and practitioners on how to consolidate ES research and practice on environmental benefits and burdens with intra- and intergenerational justice in socio-political interventions. For example, the design of payment for ecosystem services (PES) strategies for water regulation and provision of recreation opportunities in Chile included either single ecological or multiple social and ecological goals, leading to different effects and trade-offs that require an understanding of the local context to evaluate its ability to address social equity concerns (Benra et al. 2022). These benefits and burdens are distinct from the notion of disservices from nature in that they emerge from conservation, restoration or management and may entail opportunity costs as an outcome of individual or collective actions (Nelson et al. 2020).

In this paper, we approach the need for guidance by first outlining the missing link between ES and justice. We concentrate explicitly on ES assessments given that they have been elevated as a practical approach to linking environmental and human well-being (Daily and Ruckelshaus 2022), yet continue to be critiqued for lacking a clear consideration and incorporation of justice (Jax et al. 2013; Kolinjivadi et al. 2015). We apply an environmental justice framework to illuminate potential synergies between assessment and justice goals. More specifically, we highlight the currently under-researched potential to facilitate pluralism through integrating environmental justice in ES assessments, management, and research (Chan and Satterfield 2020).

## Shedding light on justice in ecosystem services research

The past decades have seen increasing attention on distributive and procedural justice aspects in ES assessments, management, and research (Table 1; Pascual and Howe 2018). This has come through a shift from framing ES as biophysical conditions and flows toward an understanding that ES are co-produced by people and nature (Palomo et al. 2016; Bruley et al. 2021) and recognition of the central role of governance as a mediator between biophysical conditions and human well-being (Primmer et al. 2015; Nunan et al. 2021; Isaac et al. 2022). However, despite an augmented interest in social-ecological systems in which ES are embedded, and despite sophisticated and diverse theoretical understandings of justice (Schreckenberg et al. 2018), explicit incorporation of justice concerns to foster more balanced and just outcomes remains a challenge both in ES research and practice (Dawson et al. 2018; Langemeyer and Connolly, 2020).

**Table 1:** Core elements of an environmental justice framework (Sikor et al 2014; Svarstad and Benjaminsen 2020)

Core element of environmental justice framework	Definition	Example questions
Recognition justice	Acknowledgement of the diversity of stakeholders, elimination of cultural domination of some stakeholders	How do a variety of actors perceive ES and human-nature relationships? (disaggregation of stakeholders)
Procedural justice	Participation of all stakeholders and rights holders in ES interventions and roles in decision making	How are decisions over ES being made? Who is involved in decision-making (governance)
Distributive justice	Distribution of benefits and costs among stakeholders, or rights and responsibilities, from ES or in ES interventions	Who is (or has been) affected positively or negatively by changes in ES supply or access to ES due to an intervention? (consequences)

An analysis of the regional and global assessments of IPBES found that key justice aspects, such as formal institutions (e.g., laws) and informal institutions (e.g., social norms, cultural preferences) that influence the distribution and the recognition of different worldviews through Indigenous and local knowledge (Martin et al. 2016) remain important shortcomings in ES research. At the same time, the relevance of these questions has increased in the IPBES assessments as compared to the Millennium Ecosystem Assessment as the environmental crisis has increased between the assessments (Mastrángelo et al. 2019; Persson et al. 2022). For example, practicing justice and inclusion in nature conservation was identified as a leverage point toward sustainable pathways in the IPBES Global Assessment, including procedural and restorative elements (Chan et al. 2020). The IPBES Regional Assessment for Europe and Central Asia found overall limited knowledge on distributive and recognitional justice concerning ES (Martin-López et al. 2018). The current inclusion of justice issues in ES research and practice remains fragmented (Friedman et al. 2018) and mainly relates to different policy instruments such as PES (McDermott et al. 2013), protected areas (Schreckenberg et al. 2016) or REDD + (Mathur et al. 2013).

If included, social justice and equity concerns in ES research and practice tend to primarily focus on the inequitable distribution of benefits and burdens from ES (Luck et al. 2012; Mandle et al. 2016; but see Gould et al. 2020). For example, some research addresses inequities in the design and implementation of PES (Kolinjivadi et al. 2014; Loft et al. 2017) and the effects this may have on the motivation to comply with PES rules (Chan et al. 2017; Law et al. 2017; Loft et al. 2020). Other studies have focused on the inequitable distribution of the potential supply of ES (Mandle et al. 2016; Felipe-Lucia et al. 2022), or have characterized barriers in access to ES for different members of society, including vulnerable and marginalized populations (e.g., Wieland et al. 2016) or specific demographic groups (Cortinovis and Geneletti, 2018). Examples include the distribution of access to urban green spaces (Nyelele and Kroll 2020), the distribution of the potential supply of ES in rural social-ecological systems and rural properties (Benra and Nahuelhual, 2019; Atkinson and Ovando, 2021), and the (unequal) use of protected areas by different groups (Booth et al. 2010).

Several studies show conceptual links between ES and environmental justice (McDermott et al. 2013; Sikor et al. 2014) by emphasizing the importance of considering other dimensions of justice beyond distribution (Langemeyer and Connolly 2020). Particularly, studies on PES increasingly analyze a variety of justice dimensions based on empirical case studies (Corbera et al. 2007; Meza Prado et al. 2021). Indigenous scholar-led work has also adapted the concept of ES to a relational framing that more adequately brings the perspectives, values, and world views of Indigenous communities to the table in natural resource management and decision making (Pascua et al. 2017; Gould et al. 2020; Winter et al. 2020). However, only a few studies have so far empirically linked environmental justice to the production and access to ES (Berbés-Blázquez et al. 2017). This happened primarily through the analysis of trade-offs between different actors´* wishes and needs as well as through investigating differences in access to and distribution of ES benefits (Dawson et al. 2017; Chaudhary et al. 2018; Turkelboom et al. 2018).

To a minor extent, work on ES and justice has broadened to include work scrutinizing the formal or informal institutions (e.g., rules, norms, laws) that structure interactions between societal and political actors, guide resource-use decisions that influence ES production and access (Kooiman, 2003). We also observe a trend toward shedding light on the interface between environmental justice and the supply side of ES (Benra and Nahuelhual, 2019; Ramirez-Gomez et al. 2020; Atkinson and Ovando 2021), as well as on equity in programs and policies, e.g., for planning green infrastructure in cities (Hoover et al. 2021). To date, ES assessments only marginally touch on the disaggregation of beneficial and detrimental ES by different groups of people and their cultural worldviews (Brück et al. 2022). Frequently neglected aspects include values, rights, responsibilities (Chan et al. 2017), capabilities (that is, the combination of a person's abilities and political, social, and environmental opportunities to choose and to act (Polishchuk and Rauschmayer 2012; Forsyth, 2015)), and the question of whose values are articulated by research programs (Vatn 2009). Another important omission in ES and equity assessments is the ignorance of power relations in decision-making (Boillat et al. 2020), especially when incompatible interests of stakeholders and rights holders, ranging from public to private ones occur (Berbés-Blázquez et al. 2016).

## The environmental justice framework as an eye-opener on systemic shortcomings

**Fig. 1:**
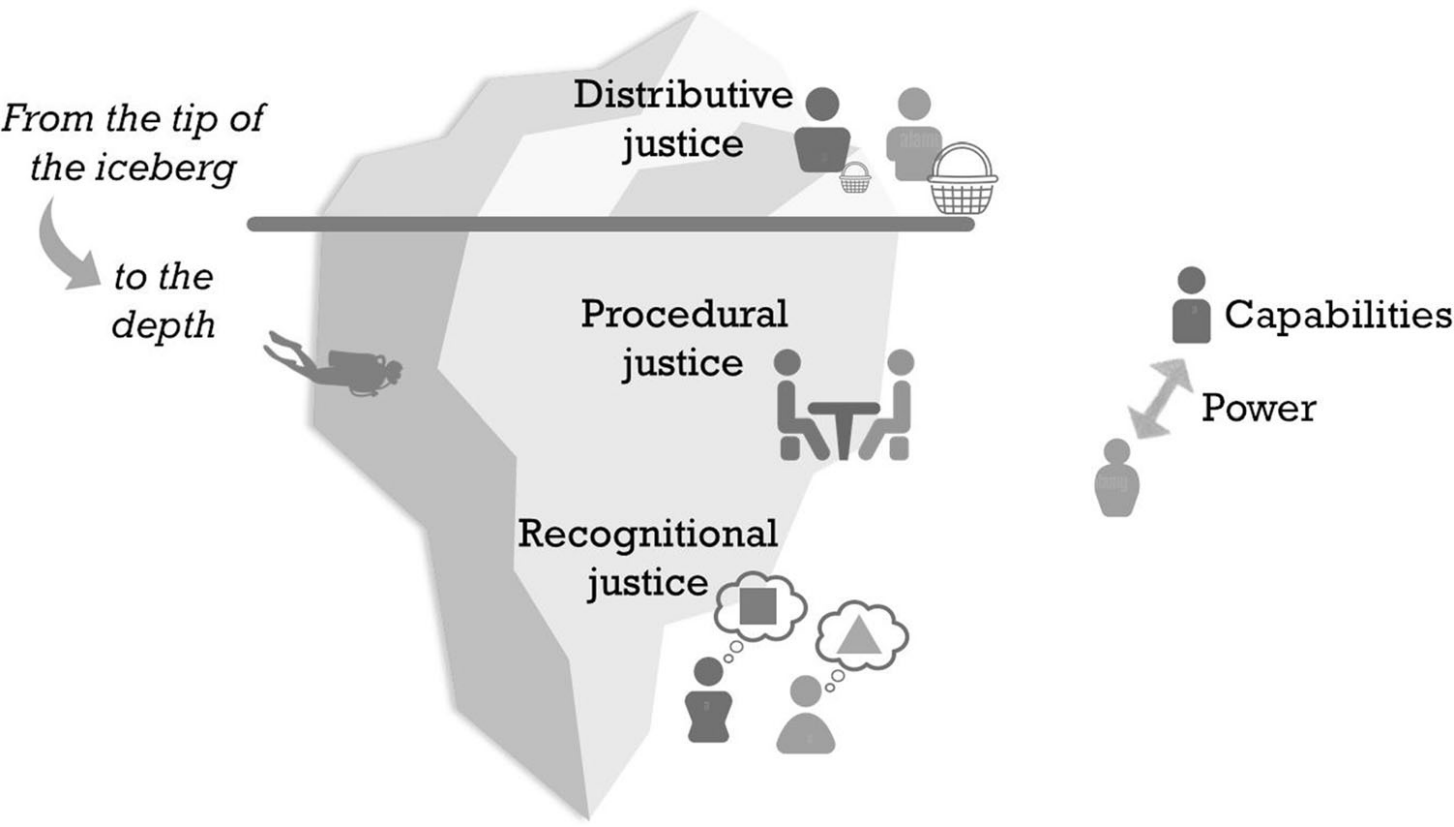
The justice dimensions intersecting with ecosystem services assessments and management displayed as an iceberg. Rather than measuring the visible, distributive dimension only, we highlight the need to ‘dive deeper’ into the social-ecological system to understand and recognize the value and knowledge plurality as well as capabilities and power structures that underpin the processes of decision-making over ecosystem services

We encourage the incorporation of the tri-dimensional environmental justice framework (Schlosberg 2004) that includes i) the recognition of actors and their respective values, rules, knowledge, and capabilities, ii) the procedure of value attribution and governance of decision-making over ES, and iii) the distribution and disaggregation both of benefits and burdens related to ES production, provision, governance, and management. By scrutinizing ES research and practice through an environmental justice lens, we suggest ways to engage both with biophysical structures, processes, and functions and their contributions to human well-being, including feedback loops and mediating factors. The justice dimension of recognition is a feasible entry point toward widening ES governance and management for diversity in powers, capabilities, knowledge, and values (Pascual and Howe 2018; Fig. 1).

A holistic perspective including the three justice dimensions can be an eye-opener on social-ecological system conditions that underpin governance and management of ES. Sen’s “The Idea of Justice'' (Sen 2009) claims that there may not be a blueprint for the right and wrong approaches in practice, because justice is both an outcome and a process that may be conceived differently by different communities as well as by different community members. Thus, rather than suggesting universally valid criteria, a context-specific democratic process adapting universal criteria of justice to the specific context conditions is needed to create a shared agreement on the most desired alternative to prevailing, unequal conditions. In this way, the focus on creating just allocation and participation in ES decision-making would benefit from a perspective that seeks to reduce the amount of inequity rather than striving for an ideal situation.

With this in mind, ES research and practice benefit from emphasizing how cultural and historical context determines shared understandings of justice and how these translate across scales and geographies (Forsyth 2015; Pascual et al. 2017b). For example, considering the complex and multi-layered effects of colonialism history on current policies is key for researchers, managers, and local communities working with ES on Hawai’i (Winter et al. 2020). In an urban context, one might acknowledge the imprints of systemic racism in the evolution of urban spaces and the distribution of nature in cities (Grove et al. 2018; Schell et al. 2020). These issues, in turn, may influence the proximate drivers of ES supply and use. Deeply inequitable systems of governance and exploitation simultaneously drive losses of ES and perpetuate and exacerbate inequities in recognition, process, and distribution which in some cases is leading to social-ecological traps (Cumming 2018; IPBES 2019b). Addressing structural inequalities in economics and governance is key to eradicating inequities in ES within social-ecological systems (Drupp et al. 2021). It requires and propels the transformative social change needed for sustainable pathways (Chan et al. 2020). Such a broader view on linking equity and ES research offers an opportunity to meaningfully contribute to sustainability (Schröter et al. 2017) in practice and research by considering intra- and intergenerational aspects of ES elicitation in terms of their distribution, the decision-making processes as well as their recognition. This paves a way to name and address value and knowledge plurality (Santos 2007; Zafra-Calvo et al. 2020) into governance, which may foster transformative processes (Laterra et al. 2019) if conservation interventions on ES are to be aligned with equity principles.

To overcome the gaps in ES research and practice outlined above, we call to integrate ES thinking into a more holistic view of contextual governance factors and to scrutinize governance arrangements in terms of their alignment with justice principles. The expanded environmental justice framework (Svarstad and Benjaminsen 2020) holds promise to disentangle three major questions (Table 1). Answering these questions by operationalizing the three environmental justice dimensions of recognition, distributive and procedural justice in work on ES is a great opportunity, as it offers to go beyond the material dimension and distributive aspects of ES toward a more holistic understanding of the multiple values that people relate to nature.

**Recognitional justice** is about understanding and recognizing the diversity of people's views on the issue at hand (Martin et al. 2016). We argue that recognitional justice offers an entry point toward the integration of environmental justice and ES. This pertains to ES practice and research in understanding and representing different worldviews and views of policy and management problems and their effects on nature and ES. Much of ES research and practice builds on a western-based, anthropocentric framework rooted in instrumental values, and often even pre-identified categories of ES that may not necessarily mirror people's lived realities of nature (Hansjürgens et al. 2016).

This can be improved, however, by representing diverse ways of knowing, including Indigenous views steeped in relationality and reciprocity (Raymond et al. 2013; Pascua et al. 2017; Dudgeo and Bray 2019; Whyte 2020, Winter et al. 2020), as well as including a variety of value perspectives. Further enhancement may be reached by inviting local perspectives on benefits and threats expressed in people’s terms and language and allowing these to structure ES assessments (Chan et al. 2012; Klain et al. 2014). Assessing and mapping those elements of nature with which people co-produce values (Palomo et al. 2016) may help to unravel what decision-makers should include in their considerations. Work on recognitional justice offers a nuanced view on diversity within communities (Chaudhary et al. 2018), both in terms of their capabilities as well as their relation to nature and what they perceive and treat as resources (Ausseil et al. 2022). Specifically, the notion of “nature’s contributions to people” addresses the recognition dimension of the environmental justice framework by uncovering what, and to whom, counts as valuable (Díaz et al. 2018). This broadened view represents people’s values in ways that represent their concerns and ways of thinking goes beyond measuring instrumental values. In particular, relational ways of knowing (Todd 2014; Hertz et al. 2020) are often better represented by explicitly recognizing values as preferences, principles, and virtues *about human relationships involving nature—*relational values (Jax et al. 2013; Chan et al. 2016). Many people are guided not primarily by instrumental costs and benefits, but rather by these values about relationships (Himes and Muraca 2018; Chapman et al. 2019, 2020; Gould et al. 2019). It is therefore an important step for recognitional justice that the IPBES conceptual framework and assessments include these other perspectives on values (Pascual et al. 2017a; IPBES 2019a).

Mapping actors, their values, capabilities, and their relation to nature then help to better understand **procedural justice** in the decision-making over ES. From an environmental justice perspective, people ought to be included in deciding over resource allocation, however, decision-making processes, including participatory ones, are subject to power dynamics and need to account for heterogeneous capabilities (Gustavsson et al. 2014). These **contextual factors** in which ES are governed in terms of environmental justice and inequalities (McDermott et al. 2013) comprise pre-existing political, economic, and social conditions, as well as access and abilities to supply and benefits. An assessment of the context involves exhaustive actor and power relations analysis (Felipe-Lucia et al. 2015), which includes the dynamics of interpersonal interactions between actors that allow people to express themselves freely in their way and provide fair and democratic access to information (see Box 1). Thus, we recommend deeper engagement with actors and their communities to disentangle contextual factors that moderate procedural justice outcomes on ES attribution and appropriation. Greater incorporation of narrative, place-based and Indigenous perspectives is a pathway to greater inclusion of equity and justice in ES work (Pascua et al. 2017; Gould et al. 2020; Meza Prado et al. 2021).

The unearthing of recognitional and procedural aspects, including power dynamics and a better understanding of people’s capabilities in the decision-making over ES, allows a clearer view of which resources are available to whom and how this contributes to human well-being. This includes shedding light on the **distribution** of the benefits and burdens of the supply and use of ES at a fine spatial scale and low levels of disaggregation including different value dimensions (Brück et al. 2022). For instance, the distribution of supply and use of ES and the values held by different ES producers and users might change at different spatial scales. In turn, biophysical analysis can aid researchers in unravelling patterns of deeper social, economic, and ecological injustices and getting acquainted with the context. It can also help understanding inter- and intragenerational issues, for example, availability and changes of certain ES or telecouplings between ES leading to local to global tradeoffs and synergies through time (Boillat et al. 2020). In turn, biophysical analyses can help understand historical issues like the actual distribution of natural assets as a product of past juncture points (Cumming 2018). The nature of ES and the way they can be accessed also play an important role in ES-environmental justice analyses. Accounting mechanisms and inequality measurement techniques for provisioning ES already exist, for perhaps the mapping of access to green spaces in urban settings (Geneletti et al. 2020). While many of these services traded in markets are related to consumable goods, carbon trading provides a counter-example of a public, non-rival and non-excludable good. However, many other regulating and cultural ES have been inadequately included in accounting and inequality measurement exercises (Davidson 2017).

Box 1 Relevant terms and concepts related to equity and ecosystem services (Modified from Calderon-Argelich et al. 2021 and Friedman et al. 2018

**Table:** 

Ecosystem services are the benefits humans derive from nature (MA 2005)
Nature’s contribution to people (NCP), are all the contributions, both positive and negative, of living nature (i.e., diversity of organisms, ecosystems, and their associated ecological and evolutionary processes) to the quality of life for people (Díaz et al.^2018^)
Environmental Justice—Plural set of conditions related to the fair distribution of resources, inclusive political processes, and institutionalized recognition of communities that allow for full human flourishing (Schlosberg 2013)
Justice—Justice is predicated on (1) equal right to most basic liberty compatible with that of others, (2) equalizing opportunity, and (3) aimed at benefiting the least advantaged (Guy and McCandless 2012)
Equity—Used here as the just distribution of environmental goods and burdens A multidimensional concept of ethical concerns and social justice based on the distribution of benefits and burdens, process and participation, and recognition, underpinned by the context under consideration. Sometimes used synonymously with fairness or justice (McDermott et al. 2013)
Equality—Egalitarian ideal, often in the context of distribution (e.g., Gini coefficient) (Syme 2018)
Distribution—Division of responsibilities and burdens versus rights and benefits (Sikor et al. 2014). Physical evenness characteristics of natural capital and ecosystem services
Fairness—Used here as individuals’ perceptions of justice arising from a judgment process (Graham et al. 2015). A subjective or perception-oriented notion of what is "fair", is shaped by a range of principles and considerations (e.g., representativeness, pro-poor). Also considered is the absence of envy. Sometimes used synonymously with equity. (McDermott et al. 2013)
Distributional Justice—Also known as distributive justice, refers to the equitable allocation of and access to material costs and benefits for all social groups in both spatial and temporal terms (Schlosberg 2013)
Procedural Justice—Also known as participatory justice, it refers to participatory and inclusive decision-making processes and it is linked with transparent and meaningful citizen involvement (Schlosberg 2013)
Recognitional Justice—Also known as interactional justice, it is related to interpersonal interactions that allow people to express themselves in their way, provision and access to information, and respect for different needs, values, preferences, and identities (Martin et al. 2016; Langemeyer and Connolly 2020)
Restorative Justice—Also known as reparative justice, it is based on acknowledging histories of social trauma and taking recovery measures (Aragao et al. 2016)
Contextual Justice—The broader social, governance, economic and cultural context, both past and present (e.g., power dynamics, gender, education, ethnicity, age), that influence an actor’s ability to gain recognition, participate in decision-making, and lobby for fair distribution (McDermott et al. 2013)

## Integration of value and knowledge plurality to include power and capabilities

A holistic perspective paves the way to integrate value and knowledge plurality for enhancing justice in ES and sheds light on power dimensions and capabilities. In addition, atoning for historical injustices through, e.g., decolonial environmental justice studies (Álvarez and Coolsaet 2020) may provide space to integrate distributive aspects of ES access, and recognize the diverse needs and aspirations especially for marginalized people, to use ES to live a dignified life. Including the three justice dimensions provides space to account for the burdens and responsibilities that are linked to conservation and efforts to safeguard ES (Pascual et al. 2017b). Assessments of equity in ES start already before conducting careful actor mapping (Reed et al. 2009) by uncovering differences in interests, capabilities, and power relations. Disaggregation of actors ensures to include perspectives of the most vulnerable actors in the system (Schröter et al. 2021). Mapping and assessing the vulnerability of actors helps to understand who the beneficiaries and actors are (Vallet et al. 2019); which values people assign to nature (Christie et al. 2019); how people conceptualize nature and their role within (e.g., Jax et al. 2013); to decipher the power of different actors within the study system (Felipe-Lucia et al. 2015), but also in their ability to express their interests. Integrating such a value and knowledge plurality in light of power dynamics and differing capabilities is key to enabling knowledge co-production (Norström et al. 2020).

Considering ES as a form of human–environment relationship brings the inextricable relational aspect to bear (White 2017; Chan et al. 2016). This relationship and its importance, however, vary not only between individuals in a community but is also imprinted by cultural, spiritual, and moral values. These deeply held values can be visible or invisible so careful investigations of the beneficiaries and providers of ES are needed. As an example, worldviews that put people at the center of shaping the environment may stand in contrast to ecocentric and relational understandings which include morals toward non-human entities. The development of the NCP approach addresses in part the need to recognize non-dichotomous worldviews and to move beyond instrumental definitions of ES or technical approaches to environmental management. As an understanding of divergences in worldviews requires careful investigations, we call for a broadening of our understanding of what actors are in a setting that allows for non-human subjects and more holistic objects in ES research and practice (Gould et al. 2020). In line with this novel way of giving voices to actors of all kinds, including non-human beings, awareness of the researchers´ positionality including their worldviews and power relations is crucial to facilitate discussions about justice and injustices in the assignment and the decision-making over ES. This includes the connection of different knowledge systems (Tengö et al. 2014), as well as reflections of researchers on their roles at the science-policy interface, and their attitudes regarding knowledge production and use (Crouzat et al. 2018; Vinke-de Kruijf et al. 2022) and the evaluation of their impacts (Chien 2022).

Integrating equity into ES assessments is a challenging endeavor. The reasons for this challenge lie within the complexity of system interactions across spatial and temporal scales, but also in the limitations of project design to integrate ES and equity: Ideally, an assessment would elaborate an understanding of the governance dynamics around ES to then identify effective measures to develop an alternative, more compatible approach to ES governance. Such an understanding should be compiled before any intervention takes place, but realistically, injustices can only be uncovered as outcomes of already established structures of the system. In this line of thinking, limited understanding of governance and social sciences fails to link ES and equity more broadly. Investing in recognition of ES from the onset in assessments requires a specific set of skills and engagement with actors that many projects cannot afford in terms of funding and time. Despite these difficulties, we encourage ES scholars and practitioners to accompany ES assessments and decision-making through a perspective on the interactions between social institutions to unravel insights on recognition, procedure, and distributive justice elements.

## Conclusion

Our broadened perspective supports transformative ES research and practice, which can help inform and design governance structures nourishing human agency to identify, manage, and enjoy ES for human wellbeing in a sustainable way. Integrating the inextricable linkages between environmental justice and ES in socio-political interventions creates the chance to scrutinize the governance of nations and economies and may help to target efforts toward transforming those onto a more sustainable trajectory. However, we also caution about the importance of contextual settings, as many places on Earth are governed in repressive regimes, and as a historical perspective, particularly the colonial past and present and other institutional legacies may superimpose power discrepancies. Thus, interventions for the maintenance of ES may result in unintended consequences that may increase environmental injustices. Nonetheless, through building genuine cross-sectoral partnerships, it may be possible to engage in a process that offers not only more just alternatives in ES management, but that strengthens future leadership and provides mutual learning opportunities from different world-views and knowledge. Such practical and relatively easily applicable approaches toward transforming environmentally unjust situations may help entering a process of shaping justice in ES governance. Instead of working toward an ideal state, we can improve unjust conditions through meaningful participation, which means respecting local traditions and collaboration modes.
